# Incidence of Hyponatremia in Patients With Indwelling Peritoneal Catheters for Drainage of Malignant Ascites

**DOI:** 10.1001/jamanetworkopen.2020.17859

**Published:** 2020-10-26

**Authors:** Shruti Gupta, Maria Clarissa Tio, Emily D. Gutowski, Michael S. Stecker, Ashish Verma, Shveta S. Motwani, David B. Mount, Gearoid M. McMahon, Sushrut S. Waikar

**Affiliations:** 1 Division of Renal Medicine, Department of Medicine, Brigham and Women’s Hospital, Boston, Massachusetts; 2 Harvard Medical School, Boston, Massachusetts; 3 Division of Angiography and Interventional Radiology, Department of Radiology, Brigham and Women’s Hospital, Boston, Massachusetts; 4 Dana-Farber Cancer Institute, Boston, Massachusetts; 5 Section of Nephrology, Department of Medicine, Boston University School of Medicine and Boston Medical Center, Boston, Massachusetts

## Abstract

**Importance:**

Indwelling peritoneal catheters (IPCs) are frequently used to drain tense, symptomatic, malignant ascites. Large-volume drainage may lead to hyponatremia owing to massive salt depletion. To date, no studies have examined the epidemiology of hyponatremia after placement of an IPC.

**Objective:**

To evaluate the incidence of hyponatremia after IPC placement, the risk factors associated with its development, and how it is managed.

**Design, Setting, and Participants:**

This cohort study retrospectively reviewed the medical records of 461 patients who had IPCs placed during the period between 2006 and 2016 at a tertiary care hospital in Boston, Massachusetts, of whom 309 patients met the inclusion criteria. Data analysis was performed from June to November 2019.

**Main Outcomes and Measures:**

Main outcomes were the incidence of hyponatremia (with a serum sodium level <135 mEq/L) after IPC placement, the risk factors for its development, and how it was managed. We also examined the clinical course of a subset of 21 patients with hypovolemic hyponatremia.

**Results:**

Of the 309 eligible patients with laboratory results both before IPC placement and 2 days or more after IPC placement, 189 (61.2%) were female, and the mean (SD) age was 59 (12) years. The overall incidence of hyponatremia after IPC placement was 84.8% (n = 262), of whom 21 patients (8.0%) had severe hyponatremia. The mean (SD) decrease in serum sodium level before vs after IPC placement was 5 (5.1) mEq/L and decreased by 10 mEq/L or more among 52 patients (16.8%). Patients with hyponatremia prior to IPC placement had an 8-fold higher adjusted odds of having persistent hyponatremia after IPC placement (odds ratio, 7.9; 95% CI, 2.9-21.7). Patients with hepatopancreatobiliary malignant neoplasms were more likely to develop hyponatremia (78 of 262 patients with hyponatremia [29.8%] vs 7 of 47 patients without hyponatremia [14.9%]). Hyponatremia was either unrecognized or untreated in 189 patients (61.2%).

**Conclusions and Relevance:**

Although the placement of an IPC is often a palliative measure, hyponatremia is common and is often untreated or unrecognized. Patients at highest risk, such as those with hyponatremia at baseline and those with hepatopancreatobiliary malignant neoplams, should be evaluated carefully prior to IPC placement and may warrant closer monitoring after placement. In all cases, hyponatremia should be evaluated and managed within the context of a patient’s overall goals of care.

## Key Points

**Question:**

What is the incidence of hyponatremia after the placement of an indwelling peritoneal catheter for malignant ascites?

**Findings:**

In this cohort study of 309 patients, the overall incidence of hyponatremia after the placement of an indwelling peritoneal catheter was 84.8%, yet hyponatremia was often untreated or unrecognized. Patients with hyponatremia prior to the placement of an indwelling peritoneal catheter and those with hepatopancreatobiliary malignant neoplasms were more likely to develop hyponatremia.

**Meaning:**

These finding suggest that hyponatremia is common among patients with an indwelling peritoneal catheter for malignant ascites, and at-risk patients may warrant closer monitoring.

## Teaser

This cohort study evaluates the incidence of hyponatremia after the placement of indwelling peritoneal catheters for drainage of malignant ascites and the risk factors associated with its development.

## Introduction

Malignant ascites occurs in patients with several terminal malignant neoplasms through portal vein compression from liver metastases, lymphatic obstruction, or peritoneal infiltration.^1^ Patients with symptomatic ascites often manifest with abdominal distension, dyspnea, and early satiety. Indwelling peritoneal catheters (IPCs), approved in 2005, allow for intermittent percutaneous drainage without physician supervision, affording patients greater independence and flexibility, as well as a lower risk of infection from fewer paracenteses.^2,3,4^

Hyponatremia is common in patients with cancer^5^ and can be very difficult to manage. Mild hyponatremia is associated with falls and cognitive deficits, whereas severe hyponatremia may lead to seizures and even death.^6,7,8^ Draining 1 to 2 L of malignant ascites daily via an IPC amounts to hundreds of milliequivalents of sodium loss per day, not accounting for additional losses that may occur in sweat, stool, and urine.^9^

Although IPC placement is often a palliative procedure, patients who undergo aggressive drainage can present with clinical signs and symptoms of volume depletion, including confusion, hyponatremia, and, in severe cases, hypotension and acute kidney injury.^10^ To date, there are no epidemiologic studies that have examined hyponatremia after IPC placement for malignant ascites. We aimed to address this gap in the literature by evaluating the incidence of hyponatremia and the risk factors for its development and by recommending how to best manage hyponatremia.

## Methods

### Study Population

We reviewed the medical records of patients at Brigham and Women’s Hospital who had IPCs placed for malignant ascites. The IPC registry was compiled using the Interventional Radiology Division Quality Assurance Database (ConexSys) and captured any tunneled IPCs that would have been placed by the interventional radiology team at Brigham and Women’s Hospital during the period between 2006 and 2016. A few peritoneal catheters are nontunneled catheters, and these catheters, as well as those placed by surgeons in the operating room, are not captured by this database. We collected data from first catheter placement from patients who had serial catheters. We excluded patients who had a catheter for 5 days or less prior to death or discharge to hospice and patients who did not have laboratory results checked 2 days or more after IPC placement.

We collected laboratory data through the Research Patient Data Repository, a centralized data registry within the Partners Healthcare System. We manually reviewed medical records to obtain information associated with patients’ demographic and clinical characteristics. We calculated the baseline estimated glomerular filtration rate at IPC placement using the Chronic Kidney Disease Epidemiology Collaboration equation.^11^

All protocols were approved by Brigham and Women’s Hospital’s institutional review board with a waiver of consent due to the fact that this was a retrospective review of medical records.This study follows the Strengthening the Reporting of Observational Studies in Epidemiology (STROBE) reporting guideline for cohort studies.

### Hyponatremia After IPC Placement

We recorded the most proximal serum sodium (sNa) level within 14 days of IPC placement, along with the nadir sNa level after IPC placement. Hyponatremia was defined as a nadir sNa level of less than 135 mEq/L (to convert to millmoles per liter, multiply by 1.0). Patients were further categorized as having mild to moderate hyponatremia (nadir sNa level between 120 and <135 mEq/L) and severe hyponatremia (nadir sNa level <120 mEq/L). We also delineated how many patients had a decrease of 10 mEq/L or more in their sNa level before vs after IPC placement.

Among patients with laboratory results after IPC placement, we collected clinical and laboratory data at the time of nadir sNa level. Because each ascitic drainage was variable in terms of volume and frequency, we recorded the weekly aggregate of ascitic volume drained as liters per week. We documented the presence of liver disease (defined as a bilirubin level >1.3 mg/dL [to convert to micromoles per liter, multipy by 17.104] and an international normalized ratio >1.5 or the presence of cirrhosis on imaging) as well as concurrent medication use within 7 days of the nadir sNa level, focusing on diuretics, nonsteroidal anti-inflammatory drugs, glucocorticoids, levothyroxine, salt tablets, and opioids. We recorded serum albumin levels at the time of IPC placement (ie, within 14 days of placement) and at the time of nadir sNa level. We documented whether patients were hyperglycemic at the time of nadir sNa level (glucose level >120 mg/dL [to convert to millimoles per liter, multiply by 0.0555]) and whether they had acute kidney injury, defined as a 1.5-fold increase in baseline serum creatinine level prior to IPC placement. We calculated the duration of IPC use (in days) until date of death, hospice, or removal of catheter, as well as the time from nadir sNa level until death.

We also collected data on urine electrolytes (urine sodium level and urine osmolarity), serum osmolarity, serum cortisol level, results of a cortisol stimulation test, and thyroid stimulating hormone level, if checked within 48 hours of the nadir sNa level. Two investigators (M.C.T. and S.G.) adjudicated the primary cause of hyponatremia based on serum and urine studies and physical examination findings and assessments recorded in the medical record. A patient was determined to have hypovolemic hyponatremia if the treating team documented volume depletion at physical examination and assessment (eg, orthostasis, tachycardia, hypotension, or reduced skin turgor) along with a urine sodium level of less than 25 mEq/L and urine osmolarity level greater than 100 mOsm/kg (to convert to millimoles per kilogram, multiply by 1.0).

### Statistical Analysis

Descriptive statistics were summarized as mean (SD) or median (interquartile range [IQR]) values for continuous variables, and frequency distributions were presented using percentages for categorical variables. Categorical variables and 2-group comparisons were assessed using the χ^2^ or Fisher exact test, and continuous variables were examined using *t* tests and Wilcoxon rank sum tests for pairwise comparisons. We used multivariable logistic regression to evaluate risk factors for hyponatremia after IPC placement, with covariates selected based on clinical significance. We determined the median survival from the time of IPC placement between those with and those without severe hyponatremia.

All statistical tests were 2-sided, and *P* < .05 was considered statistically significant. Statistical analyses were performed between June and November 2019 using SAS version 9.4 (SAS Institute Inc).

## Results

### Incidence of Hyponatremia Before and After IPC Placement

Of the 309 eligible patients with laboratory results both before IPC placement and 2 days or more after IPC placement (Figure), 189 (61.2%) were female, and the mean (SD) age was 59 (12) years (Table 1). Excluded patients did not differ from the overall cohort, except that they were more likely to be patients receiving palliative care who had an IPC in place for a fewer number of days prior to death or discharge to hospice (eTable 1 in the Supplement).

**Figure.**
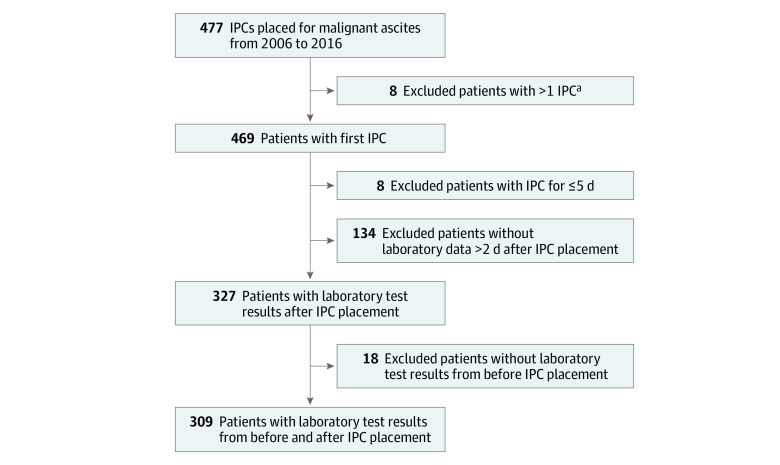
Flowchart of Enrollment IPC indicates indwelling peritoneal catheter. ^a^Data collected from first catheter placement only.

**Table 1.** Baseline Characteristics at the Time of IPC Placement

Characteristics	Patients, No. (%)			*P* value
	All (N = 309)	With hyponatremia (n = 262)	Without hyponatremia (n = 47)	
Age, mean (SD), y	59 (12)	59 (12)	68 (37)	.04
Female sex	189 (61.2)	160 (61.1)	29 (61.7)	.93
White race	252 (81.6)	219 (83.6)	33 (70.2)	.03
Type of malignant neoplasm^a^				
Breast	40 (12.9)	36 (13.7)	4 (8.5)	
Colon	22 (7.1)	18 (6.9)	4 (8.5)	
Lung	7 (2.3)	5 (1.9)	2 (4.3)	
RCC or bladder	15 (4.9)	11 (4.2)	4 (8.5)	
Cholangiocarcinoma	23 (7.4)	22 (8.4)	1 (2.1)	
Hepatocellular	6 (1.9)	6 (2.3)	0	
Ovarian	54 (17.5)	42 (16.0)	12 (25.5)	
Other gynecologic	27 (8.7)	21 (8.0)	6 (12.8)	
Pancreatic	56 (18.1)	50 (19.1)	6 (12.8)	
Unknown primary	7 (2.3)	7 (2.7)	0	
Other	48 (15.5)	40 (15.3)	8 (17.0)	
>1 Type of cancer	4 (1.3)	3 (1.1)	1 (2.1)	
Before IPC placement, mean (SD)				
Sodium, mEq/L^b^	134 (4)	134 (4)	138 (4)	<.001
eGFR, mL/min/1.73 m^2^^c^	89 (62-106)	90 (65-107)	69 (41-99)	.01
BMI, median (IQR)^d^	25 (21-28)	24 (21-27)	26 (22-29)	.02
Serum albumin, mean (SD), g/dL	2.6 (0.4)	2.6 (0.5)	2.5 (0.4)	.52
Liver disease^e^	92 (29.8)	81 (30.9)	11 (23.4)	.32

Abbreviations: BMI, body mass index (calculated as weight in kilograms divided by height in meters squared); eGFR, estimated glomerular filtration rate; IQR, interquartile range; IPC, indwelling peritoneal catheter; RCC, renal cell carcinoma.

SI conversion factors: To convert sodium to millimoles per liter, multiply by 1.0; to convert serum albumin to grams per liter, multiply by 10; and to convert bilirubin to micromoles per liter, multiply by 17.104.

^a^ A *P* value is not presented for type of malignant neoplasm given small numbers of patients within each subcategory.

^b^ Baseline sodium levels are missing for 19 patients.

^c^ Missing data on eGFR for 113 patients.

^d^ Missing data on BMI for 138 patients.

^e^ Defined as a bilirubin level of less than 1.3 mg/dL and an international normalized ratio of more than 1.5 or as the presence of cirrhosis on imaging.

The mean (SD) sNa level closest to IPC placement was 134 (5.1) mEq/L, and the mean (SD) nadir sNa level was 129 (5.2) mEq/L after IPC placement. The mean (SD) decrease in sNa level from before IPC to a nadir sNA level was 5 (5.2) mEq/L. The median duration from IPC placement to nadir sNa level was 11 days (range, 1-954 days).

Only 9 patients had severe hyponatremia before IPC placement, but 21 had a nadir sNa level of less than 120 mEq/L after IPC placement. eFigure 1 in the Supplement shows the trends in the absolute difference between sNa levels before and after IPC placement in each patient included in the analysis. Of the 309 patients, 161 (52.1%) had preexisting hyponatremia prior to IPC placement. After IPC placement, the incidence of hyponatremia was 84.8% (n = 262). Moreover, 52 patients (16.8%) had a decrease of 10 mEq/L or more in their sNa level after IPC placement. eFigure 2 in the Supplement shows trends in IPC placement, along with the incidence of hyponatremia, by calendar year from 2006 to 2016.

### Baseline Clinical Characteristics of Patients Who Developed Hyponatremia After IPC Placement

Patients who developed hyponatremia after IPC placement were younger (mean [SD] age, 59 [12] years vs 68 [37] years), had a lower body mass index (BMI; calculated as weight in kilograms divided by height in meters squared) (median [IQR], 24 [21-27] vs 26 [22-29]), and had a higher estimated glomerular filtration rate (median [IQR], 90 [65-107] mL/min/1.73 m^2^ vs 69 [41-99] mL/min/1.73 m^2^) at baseline compared with patients who did not develop hyponatremia (Table 1). They were more likely to have liver disease (81 of 262 [30.9%] vs 11 of 47 [23.4%]) and hepatopancreatobiliary malignant neoplasms (eg, 78 of 262 patients with hyponatremia [29.8%] vs 7 of 47 patients without hyponatremia [14.9%]).

### Clinical Characteristics of Patients at Nadir sNa Level and Risk Factors for Hyponatremia

At the time of nadir sNa level, patients with hyponatremia had more fluid removed via IPC each week than did patients without hyponatremia, although the difference was not statistically significant (median [IQR], 7 [4-9] L/wk vs 5 [3-6] L/wk; *P* = .13) (Table 2). Patients with hyponatremia were also more likely to be hyperglycemic (95 of 262 [36.3%] vs 11 of 47 [23.4%]) and receiving diuretics (38 of 262 [14.5%] vs 6 of 47 [12.8%]), levothyroxine (30 of 262 [11.5%] vs 1 of 47 [2.1%]), nonsteroidal anti-inflammatory drugs (12 of 262 [4.6%] vs 1 of 47 [2.1%]), and opioids (125 of 262 [47.7%] vs 21 of 47 [44.7%]). eFigure 3 in the Supplement shows the absolute difference in sNa and serum creatinine levels before vs after IPC placement. Patients who developed hyponatremia after IPC placement had a median increase in serum creatinine level from 0.70 mEq/L at the time of IPC placement to 1.0 mEq/L at the time of nadir sNa level. In contrast, those without hyponatremia after IPC placement had a median serum creatinine level of 1.12 mEq/L at IPC placement and 0.92 mEq/L at nadir sNa level. Of the 262 patients who were hyponatremic, 67 (25.6%) had acute kidney injury compared with 7 of the 47 patients (14.9%) without hyponatremia (*P* = .11).

**Table 2.** Clinical Characteristics of Patients at the Time of Nadir Sodium Level

Characteristics	All (N = 309)	With hyponatremia (n = 262)	Without hyponatremia (n = 47)	*P* value
Duration of IPC, median (IQR), d	35 (18-79)	37 (20-80)	28 (12-59)	.04
Fluid removed, median (IQR), L/wk^a^	6 (4-8)	7 (4-9)	5 (3-6)	.13
Serum albumin, mean (SD), g/dL	2.4 (0.5)	2.4 (0.5)	2.5 (0.5)	.21
Hyperglycemia, No. (%)^b^	106 (34.3)	95 (36.3)	11 (23.4)	.10
Medication use, No. (%)^c^				
Diuretic use	44 (14.2)	38 (14.5)	6 (12.8)	.71
Levothyroxine	31 (10.0)	30 (11.5)	1 (2.1)	.04
NSAIDs	13 (4.2)	12 (4.6)	1 (2.1)	.69
Opioids	146 (47.2)	125 (47.7)	21 (44.7)	.61
Glucocorticoids	83 (26.9)	68 (26.0)	15 (31.9)	.46
Salt tablets	6 (1.9)	5 (1.9)	1 (2.1)	.90

Abbreviations: IPC, indwelling peritoneal catheter; IQR, interquartile range; NSAIDs, nonsteroidal anti-inflammatory drugs.

SI conversion factors: To convert serum albumin to grams per liter, multiply by 10; and to convert glucose to millimoles per liter, multiply by 0.0555.

^a^ Missing data on weekly fluid removal for 52 patients.

^b^ Defined as glucose level greater than 120 mg/dL.

^c^ Missing data on medication use for 44 patients.

In multivariable analyses (Table 3), hepatopancreatobiliary malignant neoplasms were associated with a 5.1-fold higher odds of developing hyponatremia after IPC placement (95% CI, 1.1-24.8). A higher BMI was associated with a lower risk of hyponatremia. A lower sNa level before IPC placement was associated with higher odds of persistent hyponatremia after IPC placement (odds ratio, 7.9; 95% CI, 2.9-21.7).

**Table 3.** Risk Factors for Hyponatremia After IPC Placement^a^

Variable	Odds ratio (95% CI)	
	Univariable analysis	Multivariable analysis
Aged >60 y	0.58 (0.31-1.09)	0.60 (0.23-1.57)
Female sex	0.97 (0.51-1.84)	
Liver disease	1.44 (0.70-2.98)	
Pancreatic, liver, or gallbladder cancer	2.42 (1.05-5.60)	5.09 (1.05-24.80)
BMI (continuous)	0.90 (0.83-0.98)	0.90 (0.82-0.99)
Before IPC placement		
Sodium level <135 mEq/L	6.88 (3.39-13.95)	7.87 (2.86-21.70)
eGFR >60 mL/min/1.73 m^2^	0.37 (0.15-0.94)	
AKI at time of sodium nadir	1.99 (0.85-4.65)	

Abbreviations: AKI, acute kidney injury; BMI, body mass index; eGFR, estimated glomerular filtration rate; IPC, indwelling peritoneal catheter.

^a^ Hyponatremia was defined as a nadir serum sodium level of less than 135 mEq/L (to convert to mmol/L, multiply by 1.0).

### Treatment of Hyponatremia

Of the 262 patients with hyponatremia after IPC placement, 189 (61.2%) were untreated for their hyponatremia or their treatment data were not recorded (eFigure 4 in the Supplement). Among those who were treated, the most common treatment was intravenous fluid (IVF) (75 [28.6%]), followed by fluid restriction (10 [3.8%]). Some patients were started with diuretics (9 [3.4%]). Nephrology was consulted to assist with the management of hyponatremia for 23 patients (8.8%).

### Survival

Patients with severe hyponatremia had a median survival of 35 days (IQR, 22-104 days) from the time of IPC placement, whereas those with sodium levels of 121 mEq/L or more had a median survival of 38 days (IQR, 19-109 days).

### Subset of Patients With Hypovolemic Hyponatremia

Of the 262 patients who developed hyponatremia after IPC placemnt, only 57 (21.8%) had urine studies done. Twenty-one of the 57 patients (36.8%) were adjudicated to have hypovolemic hyponatremia based on medical record review (eTable 2 in the Supplement). For the remaining 36 patients, other causes included syndrome of inappropriate antidiuretic hormone (SIADH) and/or liver failure, although, in the majority of cases, the cause could not be determined. At the time of nadir sNa level, 16 of 21 patients (76.2%) were prescribed opioids, and 5 (23.8%) were taking nonsteroidal anti-inflammatory drugs. All patients had a urine sodium level of less than 25 mEq/L and a urine osmolarity level of more than 300 mOsm/kg (eTable 3 in the Supplement). For the 13 of 21 patients (61.9%) who had serum osmolarity levels measured, all were noted to be low (<280 mOsm/kg).

eFigure 5 in the Supplement shows sNa levels before and after IPC placement for each patient adjudicated to have hypovolemic hyponatremia and the effects of interventions to increase sNa levels. None of the patients were managed with fluid restriction alone. All patients received crystalloid in the form of normal saline with improvement in their sNa levels. Patients 4, 17, and 19 received aggressive IVF repletion, albumin, and blood with improvement in sNa level back to baseline. Patients 1, 2, 13, 14, and 17 received oral sodium chloride tablets, although only patient 17 achieved a higher sNa level. Patient 15 received diuretics with worsening of hyponatremia. Prior to the cessation of obtaining laboratory samples (due to death or discharge to hospice), patients 10, 13, and 14 had severe hyponatremia.

## Discussion

Our study demonstrates that hyponatremia is a common complication of IPC drainage of malignant ascites. Lower BMI, lower baseline sNa level, and hepatopancreatobiliary malignant neoplasms were each independently associated with hyponatremia. Importantly, hyponatremia after IPC placement was associated with shorter survival, yet it was often unrecognized or untreated.

Hyponatremia is common in patients with cancer and is associated with longer hospital stays and higher mortality.^7^ The increased usage of IPCs for regular ascitic drainage is an emerging risk factor for hyponatremia in patients with malignant ascites. We found that severe hyponatremia after IPC placement was associated with shorter survival compared with patients with either mild hyponatremia or eunatremia. Patients with and patients without severe hyponatremia after IPC placement had an overall short survival, which suggests that these patients may uniformly have a poor prognosis, regardless of their sodium levels. The evaluation and treatment of hyponatremia should therefore be considered within the context of a patient’s goals of care.

The proportion of patients with hyponatremia increased to 84.8% after IPC placement, with lower sNa level before IPC placement, lower BMI, and underlying hepatopancreatobiliary malignant neoplasms all independently associated with hyponatremia after IPC placement. Studies of elderly, institutionalized patients have identified lower BMI as a risk factor for hyponatremia.^12^ A low BMI may indicate poor nutritional status and/or volume depletion, and hyponatremia may ensue when this is accompanied by desalination from frequent IPC drainage. There is also a high prevalence of hyponatremia among patients with hepatocellular carcinoma and pancreatic malignant neoplasms,^13^ possibly associated with concurrent cirrhosis in patients with hepatocellular carcinoma^14^ and SIADH from chemotherapy in patients with pancreatic cancer.^13,15^ Thus, patients with preexisting hyponatremia, a lower BMI, and hepatopancreatobiliary malignant neoplasms may require closer monitoring for hyponatremia after IPC placement.

Studies exploring electrolyte abnormalities after frequent ascitic drainage have predominantly involved patients with cirrhosis, with conflicting results. Reinglas et al found that mild hyponatremia, hyperkalemia, and increased creatinine levels were common after IPC placement in refractory cirrhotic ascites,^16^ whereas Solbach et al found that hyponatremia and acute kidney injury were transient changes that eventually resolved.^17^ Data on patients with malignant ascites, however, are limited, even though the pathophysiology of ascites in these patients is entirely different from cirrhotic ascites. In cirrhosis, intraperitoneal fluid accumulation is a consequence of portal hypertension, vasodilator production, splanchnic arterial vasodilation, and subsequent sodium and fluid accumulation.^18,19^ Ascites is primarily managed with fluid and salt restriction, along with loop and potassium-sparing diuretics.^20,21,22^

With IPC placement, massive salt loss occurs with recurrent large-volume drainage of isotonic malignant ascites.^10^ Removing 2 L of ascites per day amounts to a total body loss of 250 to 280 mEq of sodium at each interval, which is more than the sodium content of a typical American diet. This accounts for 5% of the total body sodium and approximately 10% of extracellular fluid sodium. In the hyponatremic group, the median weekly fluid drainage was 7 L, equating to a loss of up to 980 mEq of sodium per week. Keeping up with these losses is difficult, and “desalination” is further exacerbated by gastrointestinal sodium losses (emesis and diarrhea) and poor solute intake (nausea and poor appetite).^10^ Thus, while patients with malignant ascites may present with abdominal distension and edema, with a similar urine electrolyte profile as with hypervolemic hyponatremia (urine osmolarity level >100 mOsm/kg amd urine sodium level <20 mEq/L), their overall sodium balance is negative. Sodium losses are compounded by increased arginine vasopressin secretion and a relative increase in total body water, which may lead to hyponatremia.

Despite the mechanistic differences between cirrhotic and malignant ascites, the latter is often inappropriately managed with diuretics, thus worsening volume depletion and salt loss.^10,23^ This was evident in the subset of hypovolemic hyponatremic patients in our cohort, in which diuretics worsened hyponatremia in patient 15. Hyponatremia secondary to salt loss from recurrent malignant ascitic drainage should be managed by the administration of isotonic IVF and/or albumin (154 mEq of sodium in 1 L of 25% albumin). In the hypovolemic hyponatremic subset of our cohort, repletion with IVF, albumin, or blood returned the sNa level back to baseline. Malignant ascites accumulates independent of volume status; thus, the administration of crystalloid does not worsen ascites. Replacement of sodium losses with regular IVF infusions equivalent to the amount of ascitic fluid drained is key to managing these patients. It is also critical to restrict consumption of hypotonic fluids, increase dietary salt content, and obtain biweekly laboratory samples from high-risk patients, if within the patient’s goals of care.

## Limitations

Our study has several limitations. Many patients were excluded because they were transitioned to hospice or died soon after IPC placement. Data on concurrent medication use or the amount of weekly fluid removal were missing for some patients. The underlying etiology or cause of hyponatremia could not be adjudicated for most patients owing to the lack of information on vital signs, physical examinations, and urine studies. We therefore could not determine the true incidence of hypovolemic hyponatremia in this cohort. We did not collect data on the rate of change of sodium levels and whether the patients had symptomatic hyponatremia. We also did not differentiate among those patients who were actively receiving treatment for their cancer and those who were receiving purely palliative care. Finally, we cannot definitively determine the underlying mechanism of hyponatremia in these patients, and multiple mechanisms were likely at play, including massive salt losses^10^ and increased arginine vasopressin secretion from either hypovolemia or SIADH. Our study cannot determine causality, and residual confounding is a possibility in all observational studies.

## Conclusions

In conclusion, we show that hyponatremia is common after IPC placement, yet often unrecognized or untreated. Identifying patients with preexisting hyponatremia and liver disease can assist with risk stratification and identifying those who might benefit from IVF infusions when clinically appropriate and if within a patient’s goals of care. A multidisciplinary approach with oncologists and nephrologists is critical in managing hyponatremia for these patients.

## Notes

Supplement. **eFigure 1.** Trends in Sodium Pre- and Post-Indwelling Peritoneal Catheter Placement **eFigure 2.** Frequency of Intra-Peritoneal Catheter Placement and Incidence of Hyponatremia Post-IPC Placement Per Year, 2006 to 2016 **eFigure 3.** Scatter Plot of Absolute Difference in Sodium vs Serum Creatinine Pre- vs Post-Indwelling Peritoneal Catheter Placement **eFigure 4.** Treatment of Hyponatremia **eFigure 5.** Changes in Serum Sodium in Patients With Hypovolemic Hyponatremia **eTable 1.** Baseline Characteristics of Patients Without Sodium Data Post-Indwelling Peritoneal Catheter Placement **eTable 2.** Characteristics of Patients with Hypovolemic Hyponatremia **eTable 3.** Characteristics of Hyponatremic Event
