# Percentage Error in Abstract and Results

**DOI:** 10.1001/jamanetworkopen.2020.28966

**Published:** 2020-11-18

In the Original Investigation titled “Incidence of Hyponatremia in Patients With Indwelling Peritoneal Catheters for Drainage of Malignant Ascites,”^1^ published online October 26, 2020, there was an error in the percentage of patients with hyponatremia mentioned in the Results section of the abstract and the text; hyponatremia was either unrecognized or untreated in 72.1% (189 of 262) patients, not 61.2% (189 of 309) of patients. This article has been corrected.^1^
