# Supplementary material for: Incidence of Hyponatremia in Patients With Indwelling Peritoneal Catheters for Drainage of Malignant Ascites
Source: JAMA Netw Open. 2020 Oct 26;3(10):e2017859. doi: 10.1001/jamanetworkopen.2020.17859 (PMC7588930; doi:10.1001/jamanetworkopen.2020.17859)
Supplement: Supplement. — eFigure 1. Trends in Sodium Pre- and Post-Indwelling Peritoneal Catheter Placement eFigure 2. Frequency of Intra-Peritoneal Catheter Placement and Incidence of Hyponatremia Post-IPC Placement Per Year, 2006 to 2016 eFigure 3. Scatter Plot of Absolute Difference in Sodium vs Serum Creatinine Pre- vs Post-Indwelling Peritoneal Catheter Placement eFigure 4. Treatment of Hyponatremia eFigure 5. Changes in Serum Sodium in Patients With Hypovolemic Hyponatremia eTable 1. Baseline Characteristics of Patients Without Sodium Data Post-Indwelling Peritoneal Catheter Placement eTable 2. Characteristics of Patients with Hypovolemic Hyponatremia eTable 3. Characteristics of Hyponatremic Event [file jamanetwopen-e2017859-s001.pdf]

Supplemental Online Content

Gupta S, Tio MC, Gutowski ED, et al. Incidence of hyponatremia in patients with indwelling peritoneal catheters for drainage of malignant ascites. *JAMA Netw Open*. 2020;3(10):e2017859. doi:10.1001/jamanetworkopen.2020.17859

eFigure 1. Trends in Sodium Pre- and Post-Indwelling Peritoneal Catheter Placement

eFigure 2. Frequency of Intra-Peritoneal Catheter Placement and Incidence of Hyponatremia Post-IPC Placement Per Year, 2006 to 2016

eFigure 3. Scatter Plot of Absolute Difference in Sodium vs. Serum Creatinine Pre- vs. Post-Indwelling Peritoneal Catheter Placement

eFigure 4. Treatment of Hyponatremia

eFigure 5. Changes in Serum Sodium in Patients with Hypovolemic Hyponatremia

eTable 1. Baseline Characteristics of Patients Without Sodium Data Post-Indwelling Peritoneal Catheter Placement

eTable 2. Characteristics of Patients with Hypovolemic Hyponatremia

eTable 3. Characteristics of Hyponatremic Event

This supplemental material has been provided by the authors to give readers additional information about their work.

© 2020 Gupta S et al. *JAMA Network Open.*

eFigure 1. Trends in Sodium Pre- and Post-Indwelling Peritoneal Catheter Placement

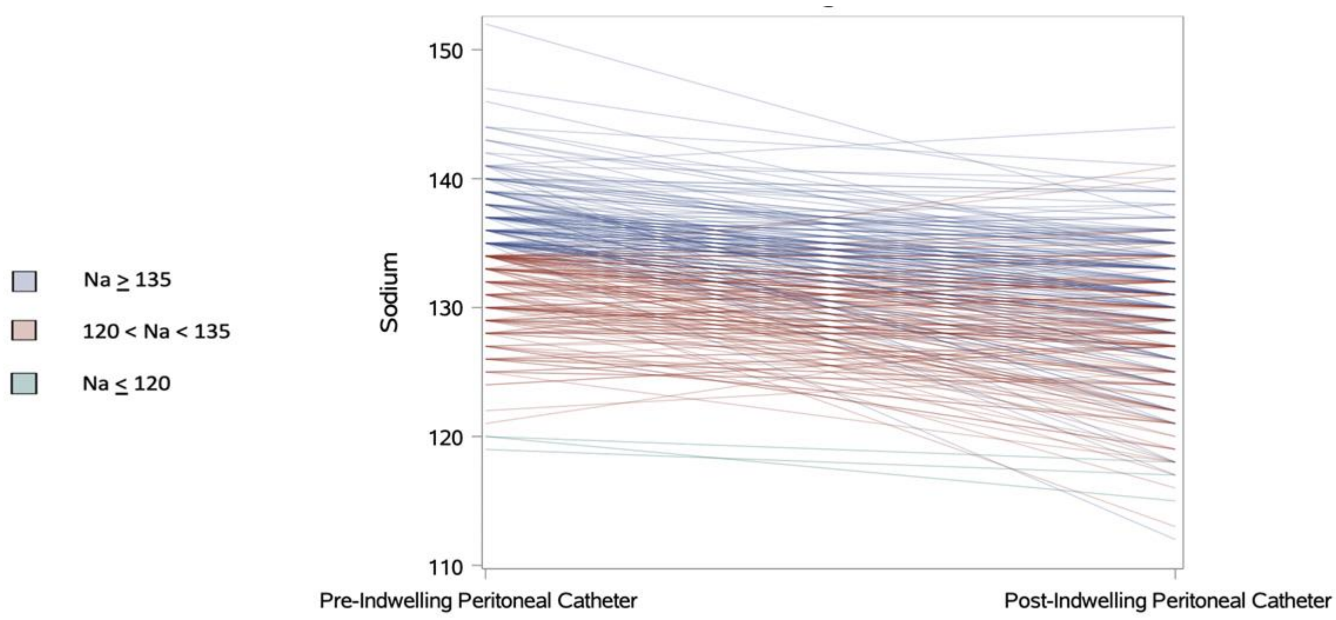

Legend:
Units: Sodium, mEq/l
Abbreviations: IPC, indwelling peritoneal catheter

© 2020 Gupta S et al. *JAMA Network Open.*

eFigure 2. Frequency of Intra-Peritoneal Catheter Placement and Incidence of Hyponatremia Post-IPC Placement Per Year, 2006 to 2016

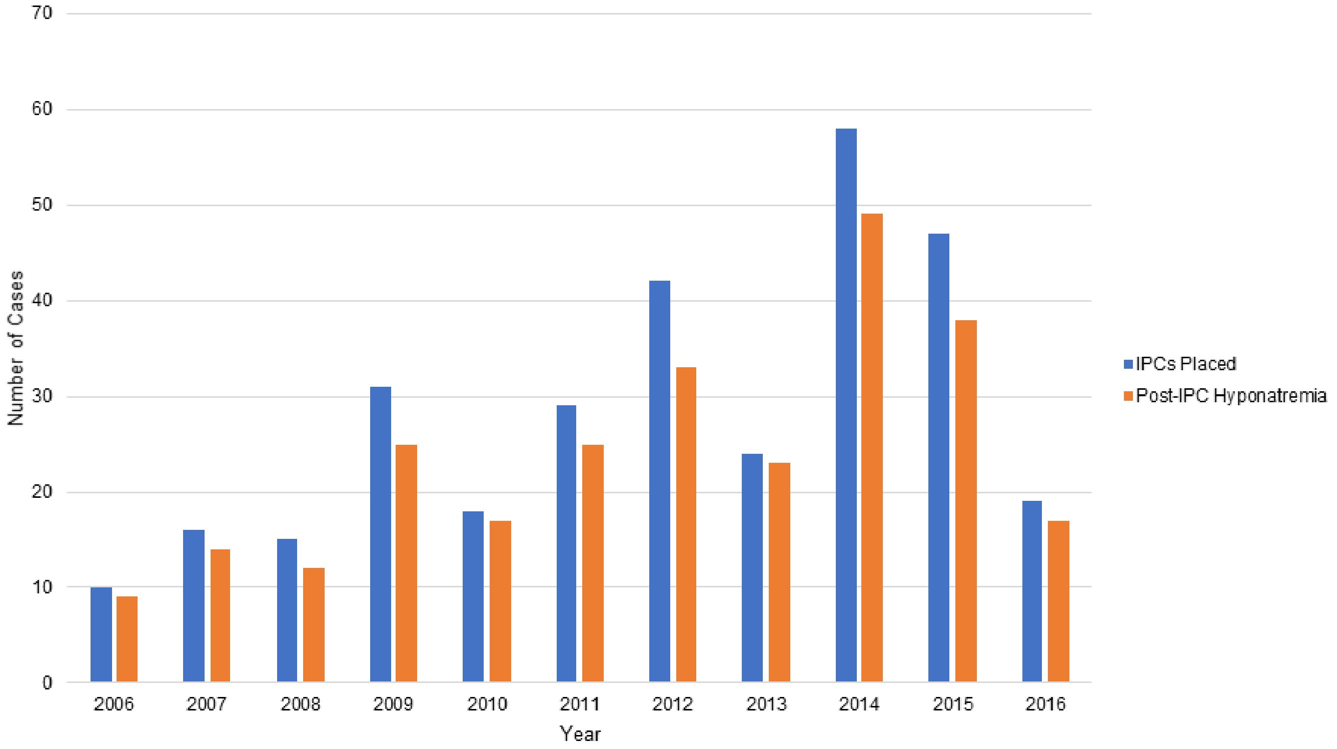

Legend:
Total n=309
Abbreviations: IPC, indwelling peritoneal catheter

© 2020 Gupta S et al. *JAMA Network Open.*

eFigure 3. Scatter Plot of Absolute Difference in Sodium vs. Serum Creatinine Pre- vs. Post-Indwelling Peritoneal Catheter Placement

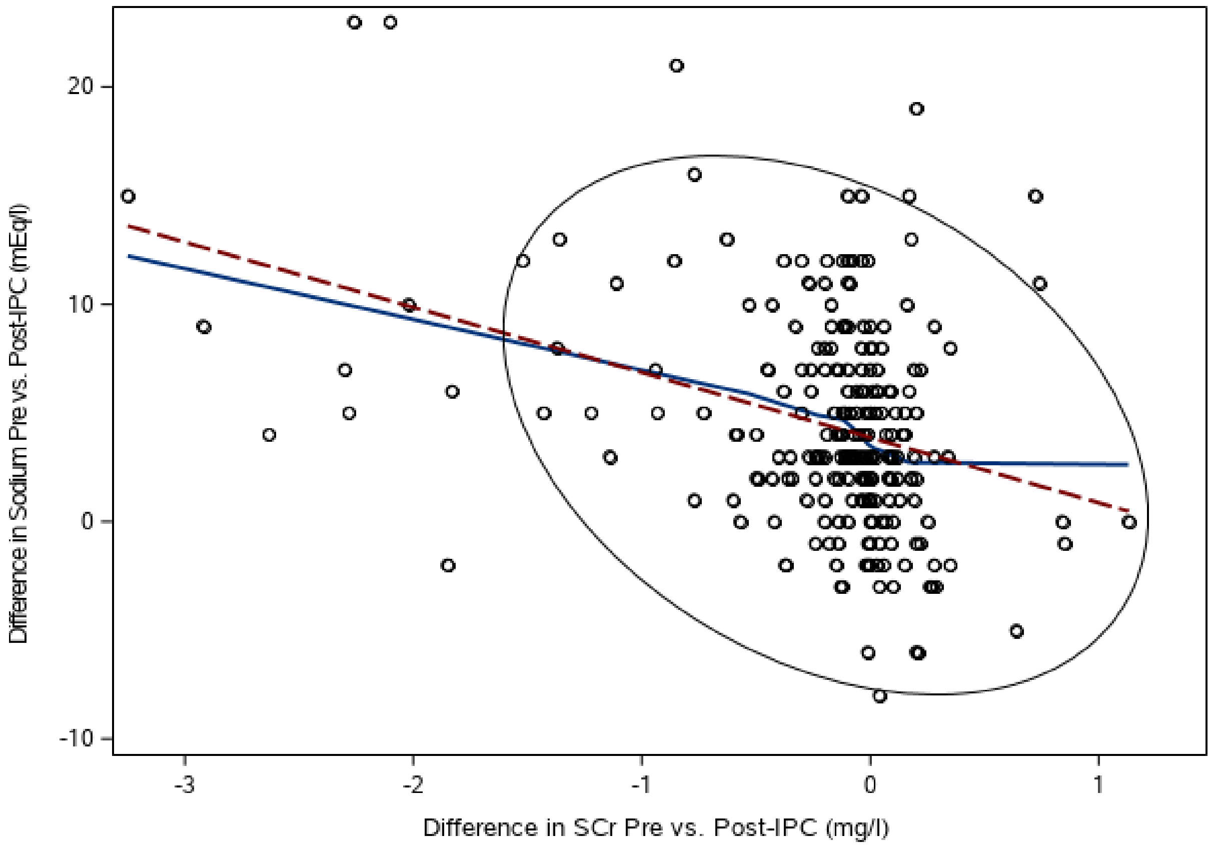

Legend:
Units: Sodium, mEq/l; sCr: mg/dl
Abbreviations: IPC, indwelling peritoneal catheter; sCr-serum creatinine

© 2020 Gupta S et al. *JAMA Network Open.*

eFigure 4: Treatment of Hyponatremia

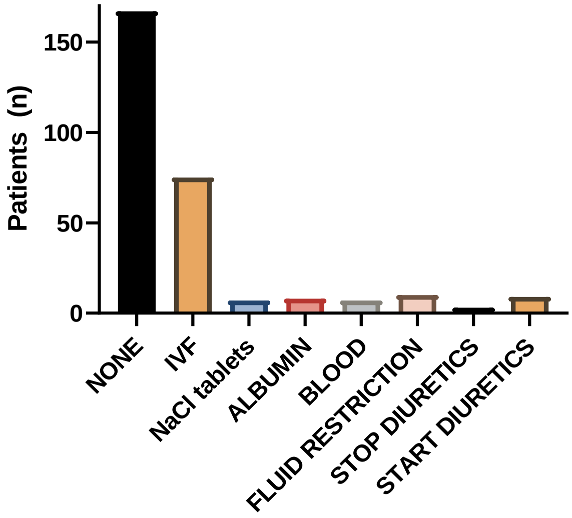

Treatment	
None/Not reported	167
Intravenous fluid	75
Salt tablets	7
Albumin	8
Blood	7
Fluid Restriction	10
Stop Diuretics	3
Start Diuretics	9
Fludrocortisone	1

Legend:
Abbreviations: IVF, intravenous fluid; NaCl, sodium chloride
Treatment of hyponatremia was determined based on manual chart review, and only at the time of sodium nadir. The amount of intravenous fluid, albumin, diuretics was not recorded, and was considered binary (yes or no) if the patient received the treatment at the time of sodium nadir.

© 2020 Gupta S et al. *JAMA Network Open.*

eFigure 5: Changes in Serum Sodium in Patients with Hypovolemic Hyponatremia

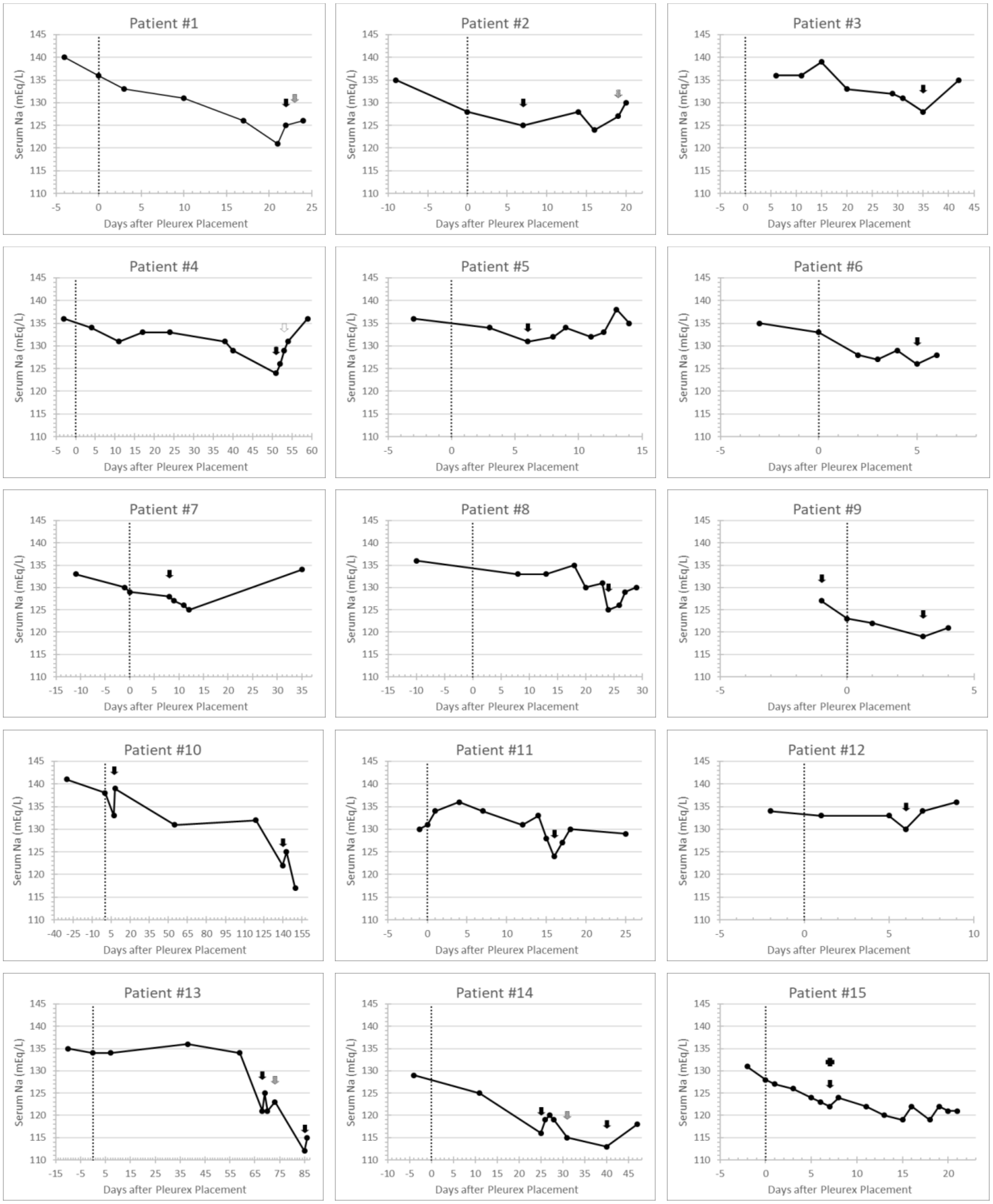

© 2020 Gupta S et al. *JAMA Network Open.*

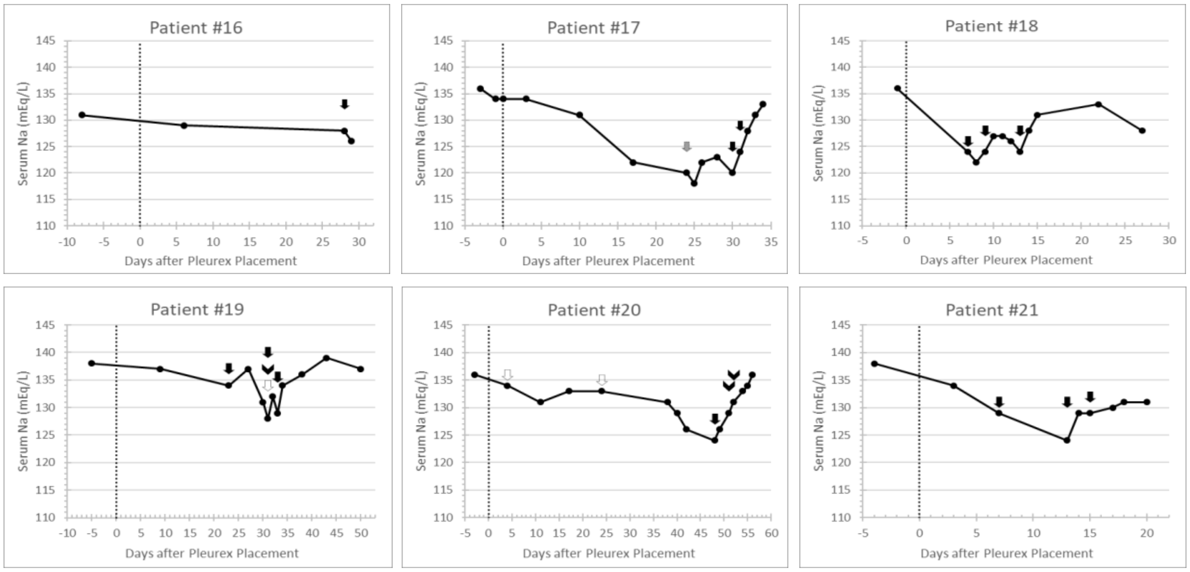

Legend: = IVF administration; = sodium administration; = blood transfusion; = diuresis; = albumin administration

© 2020 Gupta S et al. *JAMA Network Open.*

eTable 1. Baseline Characteristics of Patients Without Sodium Data Post-Indwelling Peritoneal Catheter Placement

Characteristics	Patients without Laboratory Data (n=134)
Age, mean (SD)	61 (13)
Female Sex, n (%)	72 (54)
White Race, n (%)	116 (87)
Malignancy	
Breast	13 (10)
Colon	22 (16)
Lung	4 (3)
RCC/Bladder	1 (1)
Cholangiocarcinoma	10 (7)
Hepatocellular	6 (4)
Ovarian	13 (10)
Other gynecologic	6 (4)
Pancreatic	20 (15)
Unknown Primary	3 (2)
Other	26 (19)
>1 Cancer	0 (0)
Days with IPC, median (IQR)	18 (1,35)
Liver Disease, n (%)	14 (10)
Baseline Sodium*, mean (SD)	134 (5)

Legend:
**Note: Baseline sodium not available in 78 patients*
Abbreviations: IQR, interquartile range; IPC, indwelling peritoneal catheter; RCC, renal cell carcinoma; SD, standard deviation

© 2020 Gupta S et al. *JAMA Network Open.*

eTable 2. Characteristics of Patients with Hypovolemic Hyponatremia

	Age	Sex	Race	Malignancy	Days with IPC	*Presence of Liver Disease	**Concomitant Medications at the Time of Lowest Sodium
Patient							
1	62	F	Asian	Breast	ǂ25	No	Oxycodone
2	50	M	White	Gastric	26	No	Toradol, Hydromorphone, Glucocorticoids
3	56	F	White	Uterine	50	No	Glucocorticoids, Fluoxetine
4	66	M	White	Gallbladder	107	Yes	Citalopram, Hydromorphone
5	53	F	Hispanic	Unknown	ǂ25	No	Hydromorphone, Trazodone
6	32	F	White	Colon	20	Yes	Ibuprofen, Hydromorphone
7	42	F	White	Ovarian	47	No	Ibuprofen
8	55	F	Asian/Indian	Ovarian	35	No	--
9	63	F	White	Uterine	9	No	Citalopram, Florinef, Ibuprofen, Oxycodone
10	66	M	White	RCC	180	No	Oxycodone, Oxycontin, Olanzapine
11	41	F	White	Breast	122	Yes	Citalopram, Furosemide, Spironolactone, Morphine, Sodium Chloride Tablets
12	72	F	White	Ovarian	9	Yes	Olanzapine, Oxycodone
13	48	F	White	Breast	92	Yes	Furosemide, Spironolactone, Oxycodone
14	37	M	White	Pancreas	92	No	Oxycodone, Olanzapine, Sodium Chloride Tablets
15	58	M	White	RCC	25	No	Oxycodone
16	65	M	White	Gallbladder	32	Yes	Furosemide, Spironolactone, Oxycodone
17	47	F	White	Breast	57	Yes	Olanzapine, Oxycodone, Prednisone
18	53	F	White	Uterine leiomyosarcoma	32	No	Ibuprofen
19	71	M	White	Duodenal	50	Yes	Oxycodone
20	69	F	White	Malignant mesothelioma	39	No	Olanzapine
21	66	M	White	Cholangiocarcinoma	107	Yes	Hydromorphone, Citalopram

© 2020 Gupta S et al. *JAMA Network Open.*

Legend:
[†] Patient discharged to hospice and date of death/exact number of days with IPC unknown. Number denotes days with IPC while in the hospital
** Liver disease defined as bilirubin >1.3 mg/dL and INR>1.5, or the presence of cirrhosis on imaging*
***Note: table only includes medications associated with hyponatremia or used for treatment*
Abbreviations: F, female; IPC, indwelling peritoneal catheter; M, male; RCC, renal cell carcinoma

© 2020 Gupta S et al. *JAMA Network Open.*

eTable 3. Characteristics of Hyponatremic Event

	*Pre-Existing Hyponatremia	Sodium Nadir	Amount of Ascitic Fluid Removed	TSH Levels	Urine Osmolarity	Urine Sodium	Serum Osmolarity
Patient							
1	No	121	1 to 2 L daily	---	637	<20	---
2	Yes	124	2 to 3 L daily	9.2	614	<20	263
3	Yes	128	40 mL daily	---	494	<20	---
4	No	124	2 L every other day	3.13	581	<20	276
5	No	131	1 L daily	2.55	973	<20	273
6	Yes	126	1 to 1.5 L daily	---	770	<20	271
7	Yes	125	3 L every other day	---	514	24	270
8	No	125	2 L every other day	---	348	<25	270
9	Yes	119	5 L weekly	---	620	19	265
10	No	117	1 L daily	---	387	<25	268
11	Yes	124	1.5 L daily	4.06	596	<25	---
12	Yes	130	1 to 2 L every other day	---	450	<20	---
13	Yes	112	1 L daily	---	631	<25	---
14	Yes	113	2 L daily	---	907	<20	---
15	Yes	119	2 L every other day	---	670	<10	265
16	Yes	118		---	592	<20	264
17	Yes	118	1-2 L daily	---	500	<20	255
18	No	122	1-2 L daily	2.7	899	11	264
19	No	128	1 L every other day	---	545	12	--
20	No	124	1-1.5 L twice a week	3.6	524	<10	278
21	No	124	2 L every other day	3.1	581	<20	---

Legend:
**Pre-existing hyponatremia defined as Na <135 mEq/L within 7 days of indwelling peritoneal catheter placement*
Units: sodium and urine sodium=mEq/L; urine and serum osmolarity = mOsm/kg; TSH = mIU/L
Abbreviations: L, liters

© 2020 Gupta S et al. *JAMA Network Open.*
